# 11β-Hydroxysteroid Dehydrogenase Type 1(11β-HSD1) mediates insulin resistance through JNK activation in adipocytes

**DOI:** 10.1038/srep37160

**Published:** 2016-11-14

**Authors:** Kesong Peng, Yong Pan, Jieli Li, Zia Khan, Mendi Fan, Haimin Yin, Chao Tong, Yunjie Zhao, Guang Liang, Chao Zheng

**Affiliations:** 1 Chemical Biology Research Center, School of Pharmaceutical Science, Wenzhou Medical University, Wenzhou, Zhejiang, China; 2 Diabetes Center, Department of Endocrinology, the Second Affiliated Hospital, Wenzhou Medical University, Wenzhou, Zhejiang, China; 3 Department of Pathology and Laboratory Medicine, Western University, London, ON N6A5C1, Canada

## Abstract

Glucocorticoids are used to treat a number of human diseases but often lead to insulin resistance and metabolic syndrome. 11β-hydroxysteroid dehydrogenase type 1 (11β-HSD1) is a key enzyme that catalyzes the intracellular conversion of cortisone to physiologically active cortisol. Despite the known role of 11β-HSD1 and active glucocorticoid in causing insulin resistance, the molecular mechanisms by which insulin resistance is induced remain elusive. The aim of this study is to identify these mechanisms in high fat diet (HFD) experimental models. Mice on a HFD were treated with 11β-HSD1 inhibitor as well as a JNK inhibitor. We then treated 3T3-L1-derived adipocytes with prednisone, a synthetic glucocorticoid, and cells with 11β-HSD1 overexpression to study insulin resistance. Our results show that 11β-HSD1 and JNK inhibition mitigated insulin resistance in HFD mice. Prednisone stimulation or overexpression of 11β-HSD1 also caused JNK activation in cultured adipocytes. Inhibition of 11β-HSD1 blocked the activation of JNK in adipose tissue of HFD mice as well as in cultured adipocytes. Furthermore, prednisone significantly impaired the insulin signaling pathway, and these effects were reversed by 11β-HSD1 and JNK inhibition. Our study demonstrates that glucocorticoid-induced insulin resistance was dependent on 11β-HSD1, resulting in the critical activation of JNK signaling in adipocytes.

Glucocorticoids are steroid hormones that bind to the glucocorticoid receptor (GR) and have powerful anti-inflammatory and immunosuppressive effects. However, patients treated with glucocorticoids develop obesity, insulin resistance, glucose intolerance, and dyslipidemia^1^. Currently, more than 2.5 million people in the United States are exposed to long-term glucocorticoids^2^. As such, insulin resistance resulting from glucocorticoid exposure is becoming an important public health problem and there is an urgent need to understand the mechanisms by which glucocorticoids induce insulin resistance.

Tissue-specific metabolism of glucocorticoids is catalyzed by two enzymes, 11β-hydroxysteroid dehydrogenases type 1 (11β-HSD1) and type 2 (11β-HSD2). These enzymes carry out the interconversion of non-receptor binding cortisone and the receptor binding active form, cortisol. 11β-HSD1 is an NADP(H)-dependent enzyme and converts inactive cortisone to active cortisol in the liver, adipose tissue, vasculature, and brain^3^,^4^,^5^. 11β-HSD2, on the other hand, is an NAD-dependent dehydrogenase and inactivates cortisol to cortisone in the kidney and colon^6^. The role of 11β-HSD1 in obesity and metabolic disease is well established in rodents. Transgenic mice with overexpression of 11β-HSD1 driven by the adipose tissue-specific aP2 promoter express elevated corticosterone levels in adipose tissue and display a phenotype mimicking human metabolic syndrome characterized by visceral obesity, insulin resistance, and hyperlipidemia^7^,^8^. Furthermore, administration of glucocorticoids in mice induces metabolic syndrome, which is prevented in 11β-HSD1 knockout mice^9^. These findings together with tissue-specific expression/activity of 11β-HSD1 suggest that intracellular metabolism of glucocorticoids by 11β-HSD1 is critical to the development of insulin resistance rather than the circulating glucocorticoids. Therefore, 11β-HSD1 is an important therapeutic target for reducing adverse effects of prescribed glucocorticoids for treatment of a variety of diseases

The mechanism by which increased levels of 11β-HSD1 results in insulin resistance is not fully known. The overexpression of 11β-HSD1 gene in adipose tissue can increase levels of leptin, resistin, tumor necrosis factor-α (TNF-α), and interleukin-6 (IL-6)^10^,^11^. This suggests that high local levels of glucocorticoids, at least in adipocytes, promote an inflammatory rather than the expected anti-inflammatory activity through cortisol. This inflammatory function of glucocorticoids may be regulated through c-Jun N-terminal kinases (JNK), which is believed to be a central player in the insulin signaling in diabetes and insulin resistance. Reports show that JNK knockout mice are protected against the development of insulin resistance^12^,^13^. Moreover, administration of small molecule or peptide inhibitors of JNK significantly improved insulin sensitivity in insulin-resistant rodents^14^,^15^. Multiple factors can activate JNK, such as inflammatory cytokines and free fatty acids. Importantly, glucocorticoids can also increase JNK activity in epithelial cells^16^, hippocampal cells^17^ and endothelial cells^18^. Therefore, we hypothesized that glucocorticoid-induced insulin resistance is dependent on 11β-HSD1, resulting in critical activation of JNK in adipose tissue. For study, we used the high fat diet (HFD) mouse model and cultured adipocytes to investigate this potential pathophysiological mechanism under conditions of obesity. Results indicate that glucocorticoid-induced insulin resistance was dependent **on** 11β-HSD1, resulting in the critical activation of JNK signaling in adipocytes.

## Results

### Inhibition of 11β-HSD1 and JNK improved insulin sensitivity

**Figure 1:**
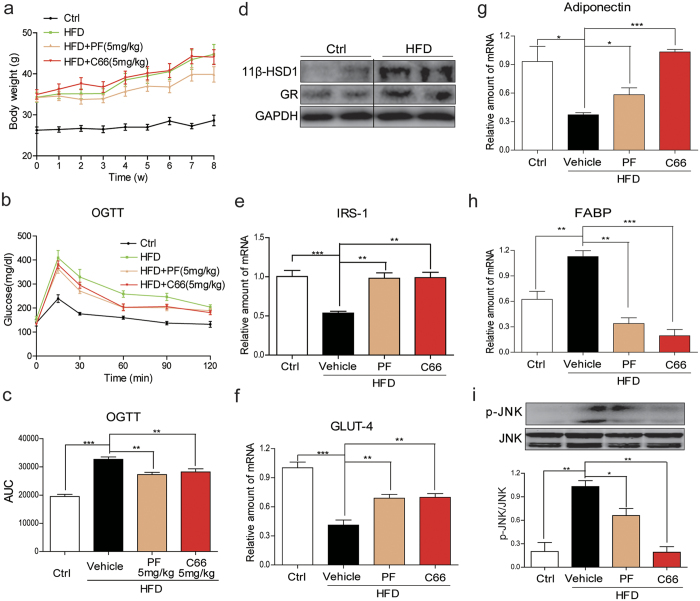
Treatment with PF00915275 and C66 inhibited insulin resistance in high-fat fed mice. PF00915275 (5 mg/kg) and C66 (5 mg/kg) were administered orally once every other day for 8 weeks to high fat diet-fed mice. (**a**) Body weight of HFD mice with or without PF00915275 or C66 treatment for 8 weeks showing that PF00915275 treatment reduced weight gain in mice fed HFD. (**b**) OGTT were performed on mice following the completion of all treatments and just before sacrifice. (**c**) Blood glucose AUC was calculated based on OGTT data presented in B. (**d**) protein levels of 11β-HSD1 and GR in subcutaneous adipose tissues from mice fed with normal chow or HFD was determined by western blot analysis. The gels were run under the same experimental conditions. Shown are cropped gels/blots (The gels/blots with indicated cropping lines are shown in Supplementary Fig. S1). Representative two mouse tissues from n = 8 were shown. (**e**–**i**) mRNA quantification of IRS-1 (**e**) GLUT (**f**) adiponectin (**g**) and FABP (**h**) by qPCR. (**i**) Akt phosphorylation levels measured in the adipose tissues. The gels were run under the same experimental conditions. Shown are cropped gels/blots (The gels/blots with indicated cropping lines are shown in Supplementary Fig. S1). Data was presented as mean ± S.E.M. *P < 0.05; **P < 0.01, ***P < 0.001 compared with the vehicle-treated group. n = 8 per group.

Specific small-molecule inhibitors of 11β-HSD1 and JNK (PF00915275^19^ and C66^20^, respectively) were used to investigate the causal mechanism between glucocorticoid excess and development of insulin resistance. Our results show that mice fed with HFD have significantly increased body weights compared to the mice fed standard chow (Fig. 1a). Treatment of HFD mice with PF00915275 reduced weight gain compared to the mice treated with vehicle after 8 weeks (39.87 ± 4.60 g vs 44.77 ± 6.78 g, Fig. 1a). As expected, blood glucose levels and glucose AUC were significantly increased in HFD mice (Fig. 1b,c). Administration with either PF00915275 or C66 significantly decreased blood glucose AUC in HFD mice compared to vehicle treatment (Fig. 1b,c). The effects of HFD on expression of 11 β-HSD1 and glucocorticoid receptor (GR) proteins in mice were next investigated. Western blot analysis showed that HFD significantly increased the expression of both 11 β-HSD1 and GR in subcutaneous adipose tissues (Fig. 1d). We also assessed the adipose tissue of PF00915275- and C66-treated mice for expression of insulin receptor substrate 1 (IRS1), glucose transporter-4 (GLUT4), adiponectin and fatty acid binding protein (FABP) as indices of insulin sensitivity. The mRNA levels of IRS-1 (Fig. 1e), GLUT4 (Fig. 1f) and adiponectin (Fig. 1g) were decreased in the adipose tissue of HFD mice, whereas treatment with PF00915275 and C66 prevented their decreased expression and thereby preserved insulin sensitivity (Fig. 1e–g). In addition, HFD upregulated the expression of FABP in subcutaneous adipose tissue which was completely abolished by PF00915275 and C66 (Fig. 1h). These data confirmed that inhibition of either 11β-HSD1 or JNK significantly attenuated the HFD-induced insulin resistance in mouse adipose tissues.

We also determined JNK activaton by measuring its phosphorylation in the mouse adipose tissues. JNK phosphorylation was significantly increased in the adipose tissue of HFD mice, which was blocked by the administration of the JNK inhibitor C66 (Fig. 1i). Importantly, treatment of the mice with the 11β-HSD1 inhibitor PF00915275 also attenuated HFD-induced JNK phosphorylation (Fig. 1a). These findings support the idea that 11β-HSD1 is an upstream regulator of JNK in modulation of insulin resistance in the HFD model.

### 11β-HSD1 is an upstream regulator of JNK activation

**Figure 2:**
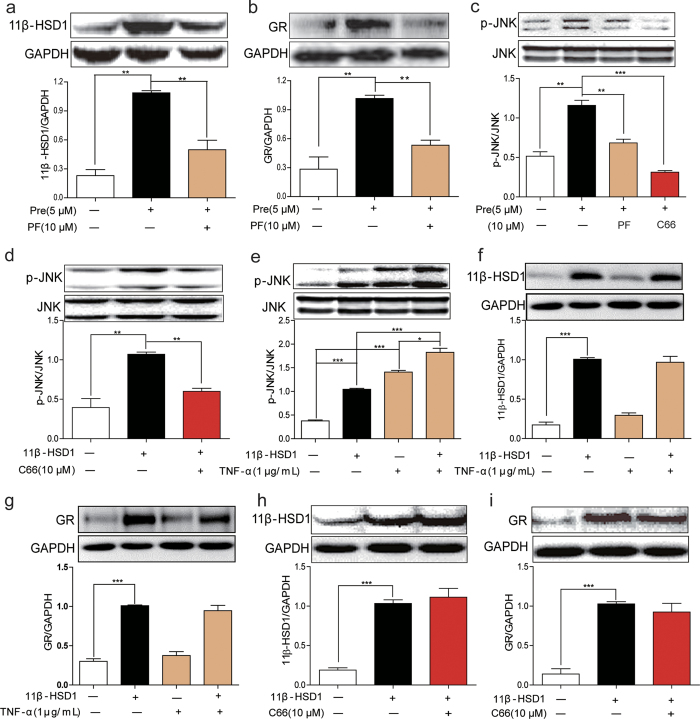
Inhibition of 11β-HSD1 reduced JNK activation in 3T3-L1 cells. (**a**,**b**) 3T3-L1 cells were pretreated with PF00915275 (10 μM) or vehicle (DMSO) for 2 h and then incubated with prednisone (5 μM) or DMSO for 1 h. Cell lysates were collected and 11β-HSD1 (**a**) and GR (**b**) were detected by western blotting. (**c**) Western blot analysis of JNK phosphorylation in adipocytes. Cells were treated as in A and B. (**d**) Cells with 11β-HSD1 overexpression were incubated with C66 (10 μM) or DMSO for 2 h. The expression of p-JNK/JNK was determined by western blot analysis. (**e**–**g**) Cells with 11β-HSD1 overexpression were incubated with TNF-α (1 μg/mL) or vehicle for 1 h. Cell lysates were collected and p-JNK/JNK (**e**), 11β-HSD1 (**f**) and GR (**g**) were detected by western blotting. (**h**,**i**) 11β-HSD1 overexpressig cells were incubated with C66 (10 μM) or DMSO for 2 h. The expression of 11β-HSD1 (**h**) and GR (**i**) was assayed. All the gels were run under the same experimental conditions. Shown are cropped gels/blots (The gels/blots with indicated cropping lines are shown in Supplementary Fig. S1). *P < 0.05, **P < 0.01, ***P < 0.001, all data from 3 independent experiments.

We further investigated the regulation of JNK by 11β-HSD1 using cultured adipocytes differentiated from 3T3-L1 cells. Treatment of adipocytes with prednisone (a synthetic glucocorticoid) resulted in increased expression of 11β-HSD1 (Fig. 2a) and GR (Fig. 2b). This increase was blocked with pretreatment of cells with 11β-HSD1 inhibitor PF00915275. As expected from our *in vivo* studies, treatment of 3T3-L1 pre-adipocytes with prednisone increased phosphorylation of JNK and this increase was abolished by PF00915275 and C66 (Fig. 2c). The findings indicated that prednisone activated a sequential pathway of increased 11β-HSD1 expression/activity and JNK activity.

We further investigated the effects of 11β-HSD1 overexpression in pre-adipocyte 3T3-L1 cells on JNK activation. Overexpression of 11β-HSD1 significantly increased JNK phosphorylation which was blocked by C66 (Fig. 2d). It is well established that TNF-α can activate JNK and induce insulin resistance. To test whether there are additive effects of TNF-α and 11β-HSD on JNK phosphorylation, we treated 11β-HSD1-overexpressing cells with TNF-α. Both TNF-α and overexpression of 11β-HSD increased JNK phosphorylation. The combination of TNF-α and overexpression of 11β-HSD further elevated the level of p-JNK (Fig. 2e). However, neither JNK activator TNF-α nor JNK inhibitor C66 altered the expression level of 11β-HSD1 and GR (Fig. 2f–i). Overall, these findings indicate that JNK is downstream of 11β-HSD1 in the regulation of insulin resistance.

### JNK inhibition by C66 attenuated prednisone- and 11β-HSD overexpression-induced insulin resistance

**Figure 3:**
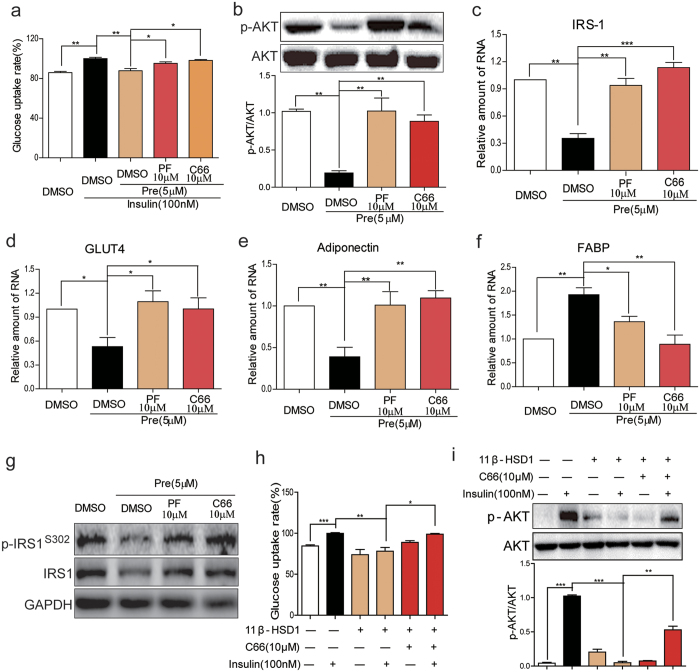
JNK inhibition by C66 attenuated insulin resistance induced by prednisone or 11β-HSD1 overexpression in adipocytes. (**a**) 3T3-L1 cells differentiated into adipocytes and then treated with PF00915275 (10 μM), C66 (10 μM) or vehicle (DMSO) for 2 h followed by incubation with prednisone (5 μM) or DMSO for 6 h. Glucose uptake rate was measured as described in the methods section. (**b**) Cells were treated with PF00915275 (10 μM), C66 (10 μM) or vehicle (DMSO) for 2 h and then with prednisone (5 μM) or DMSO for 2 h. p-Akt/Akt proteins levels were then determined by western blotting. The gels were run under the same experimental conditions. Shown are cropped gels/blots (The gels/blots with indicated cropping lines are shown in Supplementary Fig. S1). (**c**–**f**) Cells were treated as in A and mRNA levels of IRS-1 (**c**), GLUT4 (**d**), Adiponectin (**e**) and FABP (**f**) were examined by qPCR. (**g**) Cells were treated as in B and protein levels of p-IRS1^Ser302^ and IRS1 were examined by western blot analysis. The gels were run under the same experimental conditions. Shown are cropped gels/blots (The gels/blots with indicated cropping lines are shown in Supplementary Fig. S1). Representative blots from 3 independent experiments were shown. (**h**,**i**): Differentiated 3T3-L1 cells with or without 11β-HSD1 overexpression were treated with C66 (10 μM) or vehicle (DMSO) for 2 h and then incubated with insulin (100 nM) for 15 min. Glucose uptake rate (**h**) and Akt phosphorylation (**i**) were determined. The gels were run under the same experimental conditions. Shown are cropped gels/blots (The gels/blots with indicated cropping lines are shown in Supplementary Fig. S1). *P < 0.05, **P < 0.01, ***P < 0.001; all data from 3 independent experiments.

Our findings showed that the prednisone-11β-HSD1 pathway resulted in JNK activation in 3T3-L1 cells. To evaluate the significance of this JNK activation pathway in development of insulin resistance, we determined the effects of C66 on prednisone-induced insulin resistance. Treatment of 3T3-L1-derived adipocytes with prednisone decreased insulin-stimulated glucose uptake (Fig. 3a), phosphorylation of Akt (Fig. 3b), and the expression of IRS-1 (Fig. 3c), GLUT4 (Fig. 3d), and adiponectin (Fig. 3e). Moreover, prednisone also increased FABP mRNA expression (Fig. 3f). The pretreatment with JNK inhibitor C66 or 11β-HSD1 inhibitor PF00915275 significantly reversed these prednisone-induced indices of insulin resistance (Fig. 3a–f). Western blot analysis for p-IRS1^Ser302^ and IRS1 confirmed the inhibition by C66 and PF00915275 on prednisone-induced insulin resistance in 3T3-L1 cells (Fig. 3g). Consistently, the overexpression of 11β-HSD1 also decreased insulin-stimulated glucose uptake in 3T3-L1 cells, which was reversed by JNK inhibitor C66 pretreatment (Fig. 3h). Insulin increased Akt phosphorylation in control 3T3-L1 cells but not in11β-HSD1-overexpressing cells (Fig. 3i). Furthermore, pretreatment with JNK inhibitor C66 significantly reversed Akt inhibition caused by 11β-HSD1 overexpression (Fig. 3i).

### Inactivation of JNK by DN-JNK prevented the 11β-HSD1-dependent insulin resistance

**Figure 4:**
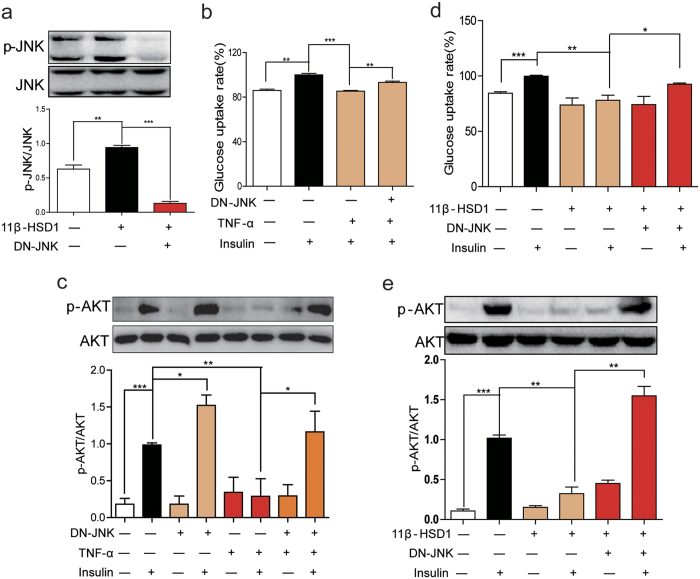
Inactivation of JNK relieved insulin resistance induced by 11β-HSD1 overexpression. (**a**) 3T3-L1 cells with or without 11β-HSD1 overexpression were transfected with DN-JNK or control plasmid and expression of p-JNK/JNK was assayed. The gels were run under the same experimental conditions. Shown are cropped gels/blots (The gels/blots with indicated cropping lines are shown in Supplementary Fig. S1). (**b**) Differentiated 3T3-L1 cells were transfected with DN-JNK or control plasmid and then incubated with TNF-α (1 μg/mL) for 6 h. Following TNF-α treatment, glucose uptake rate was detected. (**c**) Cells expressing DN-JNK were treated with TNF-α (1 μg/mL) for 15 min to assess the level of p-Akt/Akt. (**d**) 11β-HSD1 overexpressing cells were transfected with DN-JNK and incubated with insulin (100 nM) for 15 min. Following insulin treatment, glucose uptake rate was calculated. (**e**) Level of p-Akt/Akt in cells treated as in D. The gels from Fig. 4C and Fig. 4E were run under the same experimental conditions, respectively. Shown are cropped gels/blots (The gels/blots with indicated cropping lines are shown in Supplementary Fig. S1). *P < 0.05, **P < 0.01, ***P < 0.001; all data from 3 independent experiments.

To exclude possibility of any nonspecific action of C66, we generated 3T3-L1 cells expressing dominant-negative JNK (DN-JNK). As shown in Fig. 4a, transfection of DN-JNK plasmid in 11β-HSD1 overexpressing cells blocked the phosphorylation of JNK. DN-JNK also improved TNF-α- and 11β-HSD1 overexpression-induced glucose uptake (Fig. 4b,d), and insulin-induced Akt phosphorylation (Fig. 4c). Furthermore, TNF-α co-incubation with insulin inhibited insulin-induced Akt phosphorylation and this, as well, was reversed in DN-JNK-transfected cells (Fig. 4c). Lastly, we tested the effects of DN-JNK on insulin resistance induced by 11β-HSD1 overexpression. 11β-HSD1 overexpression significantly decreased insulin-induced glucose uptake (Fig. 4d) and Akt phosphorylation (Fig. 4e) in 3T3-L1 cells. As expected, blocking JNK activation by DN-JNK improved glucose update (Fig. 4d) and increased AKT phosphorylation (Fig. 4e).

## Discussion

The main finding of our study is that glucocorticoid-induced insulin resistance was dependent on 11β-HSD1, leading to JNK activation. Administration of glucocorticoids or overexpression of 11β-HSD1 in adipocytes increased JNK phosphorylation and induced features of insulin resistance, which were blocked by inhibiting 11β-HSD1 or JNK.

**Figure 5:**
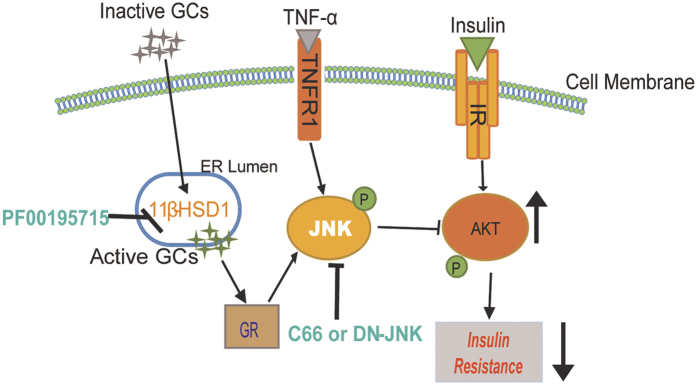
Working model depicting the underlying mechanism of 11β-HSD1- mediated insulin resistance. Glucocorticoids (inactive) cause activation of 11β-HSD1 in adipocytes producing active glucocorticoids. Active glucocorticoid-GR signaling complex activates the JNK pathway. JNK then inhibits insulin-induced Akt phosphorylation leading to insulin resistance. JNK may also be activated by inflammatory cytokines such as Tumor necrosis factor-α. [GCs = glucocorticoids, GR = glucocorticoid receptor, IR = insulin receptor, JNK = c-Jun N-terminal kinase; site of action of various inhibitors is also shown (green text)].

Glucocorticoids are widely used in the clinic as anti-inflammatory agents for a number of human diseases including autoimmune diseases, cancer, organ rejection, and asthma. However, glucocorticoids often lead to insulin resistance and diabetes, a condition referred to as steroid-induced diabetes. A number of studies have shown that the local/intracellular production of glucocorticoids by 11β-HSD1 is more critical than the circulating pool for the development of insulin resistance^9^. Over-expression of 11β-HSD1 in the mouse liver causes active glucocorticoid excess and induces metabolic disorders, while 11β-HSD1 knockout mice are resistant to HFD-induced insulin resistance and metabolic syndromes^21^,^22^,^23^. Thus, 11β-HSD1 is believed to an important target for the treatment of type 2 diabetes and metabolic syndromes. However, despite the well-documented role of 11β-HSD1 and active glucocorticoids in causing insulin resistance, the underlying molecular and cellular mechanisms remain unknown. Recent studies showed cross-talk between 11β-HSD1 and JNK, for example, that JNK activation was involved in IL-3-induced 11β-HSD1 expression in airway smooth muscle cells^24^, and inhibition of 11β-HSD1 mediated TNF-α-induced JNK activation and inflammation in pre-adipocytes^25^. However, our studies have demonstrated, for the first time to our knowledge, that JNK plays a central role in the insulin resistance induced by exogenous glucocorticoids or 11β-HSD1 activation (Fig. 5).

Adipose tissue plays an essential role in regulation of whole-body insulin sensitivity. And glucocorticoids have profound effects on the adipose tissue, such as driving central obesity by promoting the redistribution of fat from periphery to abdomen and inducing pre-adipocyte differentiation^26^,^27^. Interestingly, 11β-HSD1 is highly expressed in adipose tissues^28^. Based on the important role of 11β-HSD1 in adipose tissue and insulin resistance, we explored the 11β-HSD1-JNK pathway in regulation of insulin resistance. We found that inhibition of 11β-HSD1 or JNK was protective against development of insulin resistance in the HFD mouse model. A recent study has also shown that corticosterone treatment of adipocytes enhances 11β-HSD1 expression and impaired p-Akt leading to lipolysis. Furthermore, knockdown of 11β-HSD1 by shRNA attenuated corticosterone-induced lipolysis and improved insulin signaling response in adipocytes^29^. Taken together, these findings suggest that glucocorticoid signaling in adipocytes leads to an 11β-HSD1-dependent impairment of insulin.

A potential limitation of our study is that we did not examine the liver tissues in these mice. Insulin and 11β-HSD1 also function in the liver. However, the mechanisms may be shared with adipocytes and may also involve JNK. Whole body knockout of JNK or JNK inhibitor dramatically improved insulin sensitivity^12^. Interestingly, specific suppression of JNK activation in adipose tissue alone is sufficient to prevent the development of insulin resistance in HFD mice^30^. JNK interferes with insulin signaling through several mechanisms. JNK inhibits IRS1 function through phosphorylation of IRS1 at Ser^307 ^31 or downregulation of IRS1 expression^32^. JNK activation in adipose tissue has also been shown to decrease the production of adiponectin, which contributes to insulin resistance induced by JNK activation^30^,^33^. Furthermore, FABP is essential for adipocytes to produce TNF-α. Mice with FABP deletion are resistant to HFD-induced metabolic abnormality^34^. However, an independent group found that lipopolysaccharide induces FABP upregulation through activation of JNK^35^. Thus, FABP may also be implicated in JNK-induced insulin resistance. Our studies showed that glucocorticoid treatment or overexpression of 11β-HSD1 decreased IRS1 and adiponectin expression and increased FABP activation. These insulin resistance-related changes were reversed by JNK inhibition, establishing the importance of the glucocorticoid-11β-HSD1 pathway in JNK activation.

Activation of JNK by glucocorticoids and its involvement in insulin signaling has been also reported by other groups in different systems. For example, heat shock (DNAJB3) proteins have been shown to improve insulin signaling and glucose uptake through JNK repression^36^. Pigment epithelium-derived factor (PEDF) also suppresses JNK activation to improve hepatocyte insulin signaling^37^. We found that glucocorticoids activated JNK in adipocytes, resulting in aberrations in the insulin signaling pathway. Despite our findings and those by others, the specific molecular mechanisms by which the GC-GR complex activates JNK remain elusive. A previous study has demonstrated that GR could physically bind to JNK in dexamethasone-treated HeLa cells^38^. Our co-immunoprecipitation studies found that treatment with 5 μM prednisone for 2 h induced formation of the GR-JNK complex in 3T3-L1 adipocytes (Supplementary Figure S2), which confirmed the previous results^38^. However, the co-immunoprecipitation data could not rule out the existence of other linker proteins in the complex. In addition, multiple lines of evidence have shown that glucocorticoids can directly activate protein kinase C (PKC) isoforms^17^,^39^,^40^,^41^,^42^,^43^ which are upstream activators of JNK^44^,^45^,^46^,^47^. PKC activation has also been demonstrated to cause insulin resistance^48^,^49^,^50^. Thus, PKCs may contribute to the activation of JNK in response to glucocorticoids in adipocytes. hese observations suggest other additional mechanisms can be involved. Overall, findings from our study strongly support a role of JNK activation in GC-induced insulin resistance. An important direction for future studies is to elucidate the mechanisms by which GC/GR activate JNK.

In conclusion, our study identified a novel mechanism by which a glucocorticoid-11β-HSD1 pathway induced insulin resistance through JNK activation in adipocytes Moreover, the results suggest that 11β-HSD1 and its downstream target molecule, JNK, are key therapeutic strategies for treating insulin resistance in response to glucocorticoid excess.

## Materials and Methods

### Materials

Prednisone, 11β-HSD1 inhibitor PF-00915275^19^, TNF-α, and insulin were purchased from Sigma-Aldrich (St. Louis, MO). Compound C66, a JNK inhibitor, was synthesized and purified (>98.4%) as described in our previous studies^51^,^52^,^53^. PF00915275 and C66 were dissolved in DMSO for *in vitro* experiments and in CMC-Na (1%) for *in vivo* experiments. A 3.3-kb cDNA fragment encoding dominant negative JNK fusion protein and vector cDNA (control) were a kind gift from Professor Aimin Xu (School of Medicine, University of Hong Kong, Hong Kong)^20^. Antibodies for 11β-HSD1, GR, phospho-JNK (p-JNK), JNK, phospho-Akt (Thr308) (p-Akt), Akt and GAPDH were purchased from Santa Cruz Biotechnology (Santa Cruz, CA). The glucose concentration assay kit was purchased from Nanjing Jiancheng Bioengineering Institute (Jiangsu, China).

### Cell culture

The mouse pre-adipocyte line 3T3-L1 was obtained from the Shanghai Institute of Biochemistry and Cell Biology (Shanghai, China). Cells were cultured in Dulbecco’s modified Eagle medium (DMEM) medium (Gibco, Thermo Fisher) containing 25 mM D-glucose supplemented with 10% fetal bovine serum, 100 U/mL penicillin and 100 mg/mL streptomycin at 37 °C in a humidified 5% CO_2_ atmosphere. Upon confluence, cells were trypsinized and plated onto culture dishes for the measurement of kinase phosphorylation, and protein expression and mRNA levels. Prednisone was incubated for the indicated time intervals. For some experiments, cells were pre-treated with inhibitors for 2 h before prednisone treatment.

### 3T3-L1 cells differentiation

To induce the differentiation of 3T3-L1 cells into adipocytes, cells were cultured to confluence. Cells were then treated with complete differentiation medium which contained 10 mg/L insulin, 1 μM dexamethasone and 0.5 mM IBMX (isobutyl-methylxanthine). Medium was changed after 2 days with fresh medium containing 10 μg/L insulin, then every other day for a total of 10 days. On day 11, cells were cultured in serum-free medium for 24 h and then treated with prednisone or TNF-α for 6 h to induce insulin resistance.

### Adipocyte glucose uptake

The amount of glucose taken up by the differentiated 3T3-L1 adipocytes cells was measured by determining the difference of glucose concentrations in the culture medium before and after incubation with cells. Glucose levels in the medium were measured using a glucose assay kit. The results were expressed as glucose uptake rate compared to the insulin-treated group (100%).

### Cell transfections

3T3-L1 cells were transfected with the plasmid, pLV.EX3d.P/neo-EF1A > HSD11B1 > IRES/eGFP, to overexpress 11β-HSD1 or control plasmid using lipofectamine 2000. Cell lines were established by culturing cells in selection media with 500 μg/mLof G418.

For dominant negative JNK (DN-JNK) transfections, cells were incubated for 6 h in 1 mL serum-free medium containing 10 μL Lipofectamine 2000 and 2.5 μg DN-JNK or empty vector plasmid. Forty eight hours after transfection, cells were treated with 1 μg/mL TNF-α or vehicle control for 1 h.

### Animals

Six week old male C57BL/6 mice weighing 18–22 g were obtained from the Animal Centre of Wenzhou Medical University (Wenzhou, China). Animals were housed with a 12:12 h light–dark cycle at constant room temperature, and fed standard chow diet. The mice were acclimatized to the laboratory for at least 2 weeks before initiating studies. All animal care and experimental procedures were approved by the Wenzhou Medical University Animal Policy and Welfare Committee and performed in accordance with the approved protocols and the “The Detailed Rules and Regulations of Medical Animal Experiments Administration and Implementation”(Document No. 1998-55, Ministry of Public Health, China).

Control mice (n = 8) were fed with standard chow diet (low fat diet) which contained 10 kcal.% fat, 20 kcal.% protein and 70 kcal.% carbohydrate (MediScience Diets Co., Yangzhou, Chinca; Cat. #MD12031). The high fat diet (HFD) group was fed a diet containing 60 kcal.% fat, 20 kcal.% protein and 20 kcal.% carbohydrate (Cat. #MD12033). After 8 weeks of diet intervention, the HFD mice were randomized to 3 groups for another 8 weeks of treatment as follows: i) HFD alone group (n = 8); ii) HFD + PF00915275 (11β-HSD1 inhibitor; 5 mg/kg, orally once every other day) (n = 8); and iii) HFD + C66 (JNK inhibitor; 5 mg/kg, orally once every other day) (n = 8). Body weight measurements and oral glucose tolerance test (OGTT) were performed at the end of the treatment period. For OGTT, mice were fasted for 4 h and glucose (2 g/kg body weight) was administrated by oral gavage. Blood glucose levels were measured with a glucometer at 0, 15, 30, 60 and 120 min after glucose load. At the end of the study (week 16), the mice were anesthetized using isoflurane anesthesia and sacrificed after 12 h fasting period. The subcutaneous adipose tissue was harvested for gene and protein expression analysis.

### Real-time quantitative PCR

Cells or tissues were homogenized in TRIZOL (Thermo Fisher) for extraction of RNA. Reverse transcription and quantitative PCR were carried out using a two-step M-MLV Platinum SYBR Green qPCR SuperMix-UDG kit (Thermo Fisher). Eppendorf Mastercycler ep realplex detection system (Eppendorf, Hamburg, Germany) was used for q-PCR analysis. Primers were obtained from Thermo Fisher. The sequences were: AGGAGATGTTGGAATGACAGG (forward) and CTGAACGCTGAGCGATACATA (reverse) for Adiponectin, CATCAGCGTAAATGGGGATT (forward) and TCGACTTTCCATCCCACTTC (reverse) for FABP-4, GCCAGCCTACGCCACCATAG (forward) and AGCAGAGCCACGGTCATCAAG (reverse) for GLUT4, CAACAGCAGCAGCAGTCTTCC (forward) and CCGAGCCAGTCTCTTCTCTAGG (reverse) for IRS1, and CCGTGAAAAGATGACCCAGA (forward) and TACGACCAGAGGCATACAG (reverse) for β-actin. The amount of each gene was determined and normalized to the amount of β-actin.

### Western immunoblot analysis

Cells or tissues were lysed and 50 μg of lysate were separated by 10% SDS-PAGE and transferred to PVDF membranes. The membranes were pre-incubated for 1.5 h at room temperature in Tris-buffered saline, pH 7.6, containing 0.1% Tween 20 and 5% non-fat milk. Following primary antibody incubation, immunoreactive bands were detected by horseradish peroxidase-conjugated secondary antibodies and enhanced chemiluminescence reagents (Bio-Rad, Hercules, CA). The intensity of the bands was analyzed using Image J analysis software version 1.38e (National Institutes of Health, Bethesda, MD) and normalized to their respective control.

### Statistical analysis

The statistical significance of differences was obtained by the student’s t test or ANOVA with multiple comparisons in GraphPad Pro (GraphPad, San Diego, CA). Differences were considered to be significant at P < 0.05. Data are presented as means ± SEM.

## Additional Information

**How to cite this article**: Peng, K. *et al.* 11β-Hydroxysteroid Dehydrogenase Type 1(11β-HSD1) mediates insulin resistance through JNK activation in adipocytes. *Sci. Rep.* **6**, 37160; doi: 10.1038/srep37160 (2016).

**Publisher’s note**: Springer Nature remains neutral with regard to jurisdictional claims in published maps and institutional affiliations.

## Supplementary Material

Supplementary Information
