# Supplementary material for: 11β-Hydroxysteroid Dehydrogenase Type 1(11β-HSD1) mediates insulin resistance through JNK activation in adipocytes
Source: Sci Rep. 2016 Nov 14;6:37160. doi: 10.1038/srep37160 (PMC5107914; doi:10.1038/srep37160)
Supplement: Supplementary Information [file srep37160-s1.doc]

**Supplementary information**

**11β-Hydroxysteroid Dehydrogenase Type 1(11β-HSD1) mediates insulin resistance through JNK activation in adipocytes**

Kesong Peng^1,2^, Yong Pan^1^, Jieli Li^1^, Zia Khan^3^, Mendi Fan^2^, Haimin Yin^2^, Chao Tong^1^, Yunjie Zhao^1^, Guang Liang^1^,* , Chao Zheng^2,1^,*

1 Chemical Biology Research Center, School of Pharmaceutical Science, Wenzhou Medical University, Wenzhou, Zhejiang, China

2 Diabetes Center, Department of Endocrinology, the Second Affiliated Hospital, Wenzhou Medical University, Wenzhou, Zhejiang, China

3 Department of Pathology and Laboratory Medicine, Western University, London, ON N6A5C1, Canada

**Supplementary Figure S1**

Selected data were shown in our manuscript and the PVDF membranes for Western Blot were cut into strips to minimize the amount of antibodies that are necessary for analysis. Cropped gels/blots are presented here.

**Supplementary Figure S2**

Co-immunoprecipitation analysis of the interaction between GR and JNK.

**Figure S1.** Gels/blots with the cropping lines.


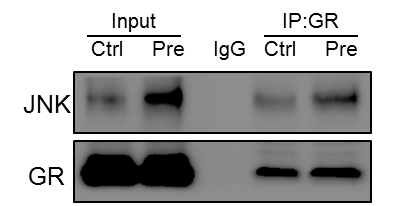


**Figure S2:** Co-immunoprecipitation analysis of the interaction between GR and JNK. 3T3-L1 cells were treated with DMSO (vehicle, Ctrl) or prednisone (5 μM) for 2h, and then the cells were lysed with lysis buffer. Cell lysates were immunoprecipitated using the GR antibodies or Rabbit control immunoglobulin G (IgG). After incubated with gentle rocking overnight at 4 ^o^C, the lysates were added protein A/G beads with gentle rocking 4h at 4 ^o^C. After extensive washing, GR and JNK protein levels in precipitates were analyzed by Western blot analysis.
